# Subthalamic nucleus deep brain stimulation in two siblings with chorea-acanthocytosis

**DOI:** 10.1007/s10072-020-04246-3

**Published:** 2020-01-14

**Authors:** Yunhao Wu, Hongxia Li, Chencheng Zhang, Bomin Sun, Dianyou Li, Yiwen Wu

**Affiliations:** 1 Department of Functional Neurosurgery, RuiJin Hospital, Shanghai Jiao Tong University School of Medicine, Shanghai, China; 2 Department of Neurology, RuiJin Hospital, Shanghai Jiao Tong University School of Medicine, Shanghai, China

Dear Editor,

Chorea-acanthocytosis (ChAc) is a core neuroacanthocytosis syndrome and a rare neurodegenerative disease that principally affects the basal ganglia. It is caused by autosomal recessive mutations of VPS13A. The characteristic clinical features of ChAc include the appearance of acanthocytes in the peripheral blood smear and mixed movement disorders including, dystonia, chorea, and parkinsonism. Some patients also have psychiatric and cognitive symptoms. Self-mutilating tongue and lip biting, which can be painful and detrimental, are unique symptoms of ChAc [1]. So far, only symptomatic treatment is available for the management of the ChAc. Oral medications and botulinum toxin injections have limited efficacy in patients with ChAc. Emerging evidence has supported the clinical benefit of deep brain stimulation (DBS) of the internal globus pallidus (GPi) for the treatment of patients with ChAc; however, sufficient beneficial results are usually observed only after several months [2]. Subthalamic nucleus (STN) DBS is an alternative to GPi-DBS for idiopathic isolated dystonia patients with the potential advantages of rapid-onset efficacy and less battery consumption [3]. Here, we present the first report of STN-DBS for the treatment of two sibling patients with drug-resistant hyperkinetic symptoms seen in ChAc, which was associated with significant as well as long-lasting efficacy.

The older sibling was a 35-year-old woman, who was admitted to our hospital with complaints of involuntary movements of the tongue, lower jaw, neck, trunk, and lower limbs. Symptoms began with sudden involuntary jaw movements and gait abnormality due to left foot varus and truncal instability at her age of 33 years. By the age of 34 years, she developed tongue and lip biting habits and was unable to adequately perform activities of daily living for increased difficulty in speaking, chewing, and swallowing. This persistent self-mutilation behavior caused her severe tongue infections as a result (video), thus forced her to stuff a towel into the mouth to prevent further injuries. Clonazepam, amantadine, and tetrabenazine had been tempted for her symptoms control but with only mild improvement. Owing to her unique symptoms (self-mutilating tongue and lip biting habit) and the appearance of acanthocytes in the peripheral blood, genetic analysis of VPS13A was conducted, which revealed c.6109delC mutation in the VPS13A gene, confirming the diagnosis of ChAc. Bilateral STN-DBS was performed after a comprehensive consideration with informed consent obtained at her age of 35, turning out to have a remarkable remission in tongue biting, dysarthria, and gait abnormality.

Eight years later, her brother was admitted to our hospital for dystonic and choreiform movements affecting the mouth, neck, and upper limbs (video). The 35-year-old man had the same clinical diagnosis, following genetic testing, which also revealed VPS13A mutation. His first symptom was oromandibular dystonia presenting as involuntary jaw movement at the age of 31 years. Progressive impairment of fine movements of the upper extremities started 2 years later, which mainly involved the right limb affecting handwriting. His treatment strategy and surgical target was the same as his sister and surgery was performed at the age of 35.

The two siblings had similar oromandibular dystonia symptoms and onset age though different initial symptom severity. We applied Burke–Fahn–Marsden Dystonia Rating scale and Unified Huntington’s Disease Rating Scale to evaluate their symptom severity of the limb and truncal dystonia and orofacial choreiform movements at baseline, stimulation test, 3-month, and 1-year follow-ups, respectively (Table 2). They have been able to resume daily activities and remain in stable conditions through the regular phone call follow-ups. The final optimized parameters were as follows: right case (+), contact3,4(−); amplitude, 2.95 V; pulse width, 50 μs; frequency, 130 Hz; left case (+), contact7,8(−); amplitude, 3.0 V; pulse width, 50 μs; frequency, 130 Hz for the brother; right case (+), contact3,4(−); amplitude, 3.15 V; pulse width, 60 μs; frequency, 160 Hz; left case (+), contact7,8(−); amplitude, 3.0 V; pulse width, 60 μs; frequency, 160 Hz for the sister. No serious adverse events occurred during the surgical and follow-up periods.

## Discussion

**Fig. 1:**
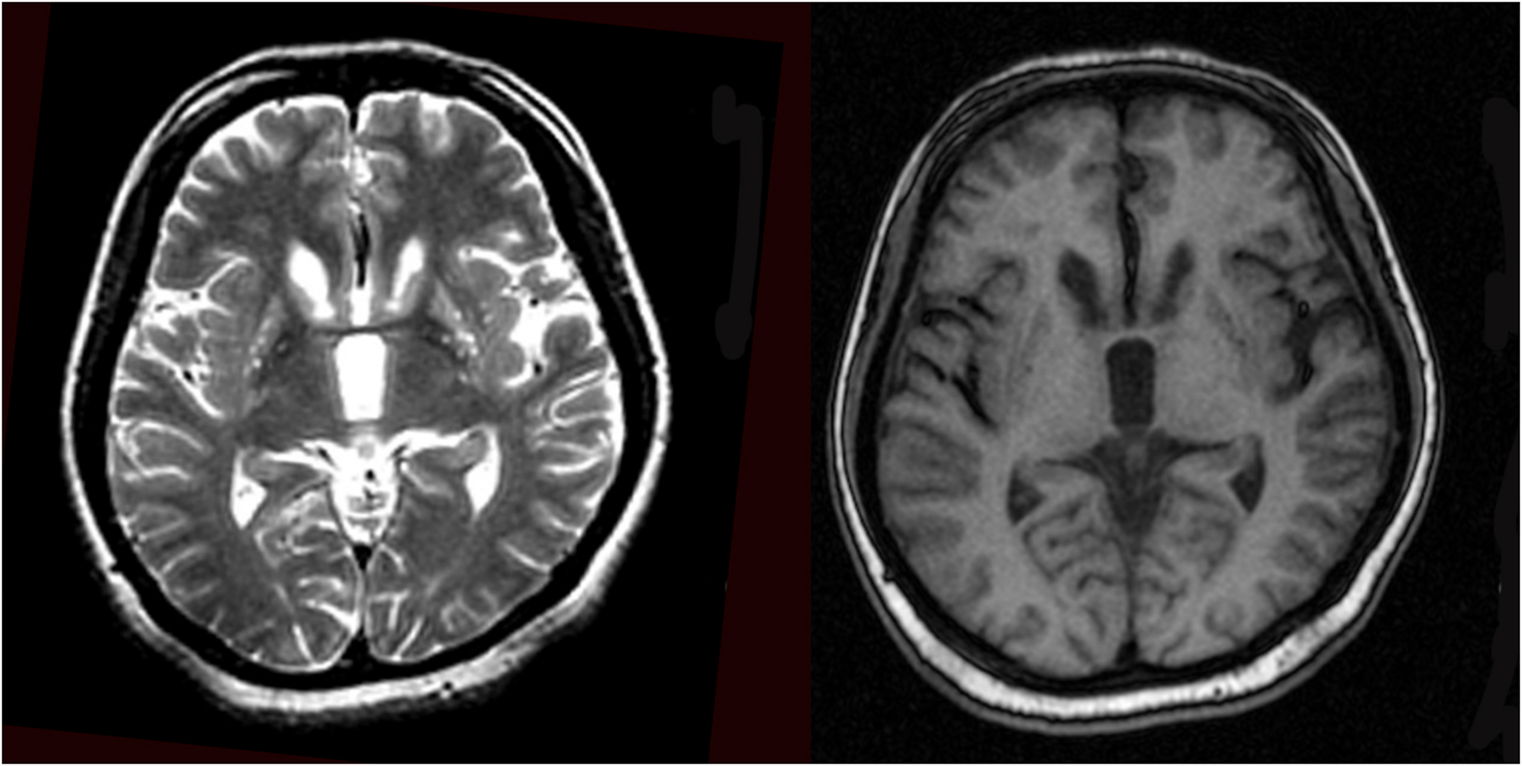
MRI of patient demonstrating bilateral striatal atrophy and degeneration

Up till now, more recent reported cases of ChAc, which were poorly controlled by conventional symptomatic drug therapy, tempted to use GPi-DBS, following the success in the management of other hyperkinetic movement disorders such as Huntington’s disease and Tourette’s syndrome [4]. GPi-DBS for ChAc patients usually presented significant and long-lasting improvement in motor disorders, according to a multi-center retrospective analysis [5] and the result of a recent 5-year review (Table 1). However, the first patient with the decision of STN-DBS was under the consultation by our multidisciplinary DBS team and the rationale considered was as follows: (A) Preoperative magnetic resonance imaging (MRI) demonstrated unexplained bilateral striatal degeneration (Fig. 1), which might affect the electrode positioning and the efficacy of GPi stimulation; (B) rapid onset and efficacy of STN stimulation [3]. Since the consideration of severe self-mutilation, rapid amelioration of oromandibular dystonia was a priority for the patient. Thus, GPi-DBS, which usually requires 1 to 6 months to be effective according to previous reports, failed to meet these criteria. The first step of DBS procedure included implanted electrodes connected to a temporary pulse generator in vitro for testing the effect of stimulation. With the efficacy of STN stimulation confirmed, subsequent permanent pulse generator implantation was completed. Fortunately, the patient showed immediate remission of tongue biting and gait improvement after surgery during the postoperation test (video). Such improvement of symptoms as well as quality of life was stabilized during long-term observation. Her brother, whose initial symptoms were less severe, also showed a comparable surgical remission of involuntary orofacial movements 8 years later (Table 2).

**Table 1:** Summary of reports/studies with ChAc treated by Gpi DBS in the past 5 years (publication year from 2014 to 2019)

Authors	Number of cases (n)	VPS13 mutation	disease(s) course(years)	Amplitude	Pulse width	Frequency	Functional outcomes
*Lee et al.* 2015 [1]	1	+	3	2.9V(Left) 2.3V (Right)	60μs	130Hz	UHDRS (motor) [59 preoperation;36 after 1 year]
Fernandez-Pajarin *et al.* 2016 [2]	1	+	7	N/A	212μs	60Hz	IPDRS (motor) [39 pre operation;13 after 1 year]
Liu *et al.* 2018 [3]	6	+	3to7	N/A	75μs to 98μs	130Hz to 175Hz	UPDRS (motor) [35.7±16.3 preoperation;13.5±5.8 after 3 months]; UPDRS (chorea)[11.3±4.7 preoperation; 3.0±1.2 after 3 months]
Wang *et al.* 2019 [4]	1	+	5	2.2V	60μs	130Hz	UPDRS (total)[61 preoperation;31 after 1 year]; UPDRS (chorea) [3 preoperation; 1 after 1 year]; BFMDRS (total) [44 preoperation; 20 after 1 year]

UHDRS: Unified Huntington’s Disease Rating Scale; BFMDRS: Burke-fahn-Marsden Dystonia Rating Scale; N/A: not available.

**Table 2:** The dystonia severity of two siblings evaluated by BFMDRS and UHDRS at baseline, stimulation test and follow-up visits

	BFMDRS								UHDRS		
	Motor Score				Disability score				Motor score		
	Baseline	Stimulation test	3-month follow-up	1-year follow-up	Baseline	3-month follow-up	1 year follow-up	Baseline	Stimulation test	3-month follow-up	1-year follow-up
Patient 1 (the older sibling)	54.5	29.5	18.5	20	16	10	10	51	30	27	28
Patient 2 (the younger sibling)	28	20	15.5	14	12	8	8	40	26	22	21

BFMDRS,Burke-Fahn-Marsden Dystonia Rating Scale: UHDRS, Unified Huntington’s Disease Rating Scale.

Here, we present the first two cases of ChAc patients treated by STN-DBS (far as we know). In these two cases, STN-DBS was effective in improving chorea symptoms, which might be attributed to the modulation of the common motor circuit, comprising the GPi and STN regions. Though the previous benefits gained from STN stimulation, it is a controversial selection for the potential risk of cognition impairment and insufficient efficacy for axial symptoms. However, for severe oromandibular dystonia and gait abnormalities symptoms of these two patients, STN-DBS seemed to possess significant advantages of rapid-onset efficacy without obvious side effects. Further controlled trials with larger sample sizes are needed to assess the efficacy of STN stimulation for different types of symptoms.

These cases indicated that STN-DBS might provide effective, long-lasting, and more rapid-acting treatment for ChAc. Owing to the lack of clinical evidence and knowledge of this rare disease, it is difficult to compare the pros and cons of GPi and STN stimulation. However, we hypothesized that STN-DBS might be better suited for the treatment of severe symptoms, such as self-mutilation behaviors, which has its treatment value for target selection. Moreover, basic clinical studies are needed to enable better understanding of the mechanisms underlying ChAc and discover ideal target and treatment modalities.

## Electronic supplementary material

ESM 1 The first fragment shows the facial and oral movements of the brother, before and after surgery. The second fragment shows the gait improvement of the sister after surgery. The third and fourth fragments show the appearance of the lip and tongue of the sister. Suppurative lesions can be seen on the tongue, due to injuries from self-mutilation behavior, before surgery; the wound gradually healed after the biting stopped. (MOV 62333 kb)
